# The epidemiological characteristics of deaths with COVID-19 in the early stage of epidemic in Wuhan, China

**DOI:** 10.1186/s41256-020-00183-y

**Published:** 2020-12-21

**Authors:** Jianjun Bai, Fang Shi, Jinhong Cao, Haoyu Wen, Fang Wang, Sumaira Mubarik, Xiaoxue Liu, Yong Yu, Jianbo Ding, Chuanhua Yu

**Affiliations:** 1 Department of Epidemiology and Biostatistics, School of Health Sciences, Wuhan University, 115#Donghu Road, Wuhan, 430071 China; 2 School of Public Health and Management, Hubei University of Medicine, 30# South Renmin Road, Shiyan, 442000 China; 3 YEBIO Bioengineering Co., Ltd. of Qingdao, 21# Aodongnan Road, Qingdao, 266114 China; 4 Global Health Institute, Wuhan University, 185# Donghu Road, Wuhan, 430072 China

**Keywords:** COVID-19, Coronavirus disease 2019, Wuhan city, Epidemiological characteristic, Death

## Abstract

**Objectives:**

To analyze the epidemiological characteristics of COVID-19 related deaths in Wuhan, China and comprehend the changing trends of this epidemic along with analyzing the prevention and control measures in Wuhan.

**Methods:**

Through the China’s Infectious Disease Information System, we collected information about COVID-19 associated deaths from December 15, 2019 to February 24, 2020 in Wuhan. We analyzed the patient’s demographic characteristics, drew epidemiological curve and made geographic distribution maps of the death toll in each district over time, etc. ArcGIS was used to plot the numbers of daily deaths on maps. Statistical analyses were performed using SPSS and @Risk software.

**Results:**

As of February 24, 2020, a total of 1833 deaths were included. Among the deaths with COVID-19, mild type accounted for the most (37.2%), followed by severe type (30.1%). The median age was 70.0 (inter quartile range: 63.0–79.0) years. Most of the deaths were distributed in 50–89 age group, whereas no deaths occurred in 0–9 age group. Additionally, the male to female ratio was 1.95:1. A total of 65.7% of the deaths in Wuhan combined with underlying diseases, and was more pronounced among males. Most of the underlying diseases included hypertension, diabetes and cardiovascular diseases. The peak of daily deaths appeared on February 14 and then declined. The median interval from symptom onset to diagnosis was 10.0 (6.0–14.0) days; the interval from onset to diagnosis gradually shortened. The median intervals from diagnosis to death and symptom onset to deaths were 6.0 (2.0–11.0), 17.0 (12.0–22.0) days, respectively. Most of the disease was centralized in central urban area with highest death rate in Jianghan District.

**Conclusion:**

COVID-19 poses a greater threat to the elderly people and men with more devastating effects, particularly in the presence of underlying diseases. The geographical distributions show that the epidemic in the central area of Wuhan is more serious than that in the surrounding areas. Analysis of deaths as of February 24 indicates that a tremendous improvement of COVID-19 epidemic in Wuhan has achieved by effective control measures taken by Wuhan Government.

**Supplementary Information:**

The online version contains supplementary material available at 10.1186/s41256-020-00183-y.

## Introduction

The outbreak of new infectious diseases in recent years has caused great losses to human health, quality of life and economy. Millions of infections were caused worldwide by Severe Acute Respiratory Syndrome Coronavirus (SARS-CoV) in 2003, Middle East Respiratory Syndrome Coronavirus (MERS-CoV) in 2012, Ebola virus in 2014, Zika virus in 2015, and the plague in Madagascar in 2017 [1–3].

In December 2019, a new coronavirus began to spread in Wuhan and even the whole country. On December 31, an alert was issued by the Wuhan Municipal Health Commission and a rapid response team was sent to Wuhan by the Chinese Center for Disease Control and Prevention (China CDC). Epidemiological investigation implicated that this pneumonia epidemic was different from the previous ones and Wuhan Huanan Seafood Wholesale Market may be a possible source of infection. On January 1, the Huanan Seafood Wholesale Market was shut down. On January 20, China’s “National Infectious Diseases Law” was amended to make 2019-novel coronavirus diseases (COVID-19) a Class B notifiable disease and its “Frontier Health and Quarantine Law” was amended to support the COVID-19 outbreak response effort. On January 23, the Chinese Government began to limit movement of people in and out of Wuhan. As of February 5, two newly established COVID-19 designated hospitals and several mobile cabin hospitals have been opened.

The WHO officially named this unexplained pneumonia as Coronavirus Disease 2019 (COVID-19) on February 11, 2020. Based on the genetic structure of the virus, the International Committee on Taxonomy of Viruses officially named the virus as Severe Acute Respiratory Syndrome Coronavirus 2 (SARS-CoV-2) [4], which is more contagious than both of the previous forms (SARS-CoV and MERS-CoV).

On March 11, the WHO officially declared the epidemic caused by COVID-19 as Pandemic. This is the first time in this century that coronavirus infection (SARS, MERS, COVID-19) has been evaluated as Pandemic.

As of February 24, 2020, 77,658 cases diagnosed with COVID-19 had been reported in Mainland China, among which 64,786 (83.42%) cases were reported in Hubei Province and 47,071 (60.61%) were in Wuhan city. Among the 2663 deaths across Mainland China, 2563 (96.24%) deaths occurred in Hubei Province and 2043 (76.72%) deaths in Wuhan city. The epidemiological features of COVID-19 cases are crucial for the development and implementation of effective control measures. In this study, we retrospectively collected and described detailed epidemiological and demographic characteristics of deaths caused by COVID-19 in Wuhan to understand the changes of the COVID-19 epidemic and the effects of prevention and control measures in Wuhan, China.

## Methods

### Data sources

As a retrospective cross-sectional study, all data were extracted from China’s Infectious Disease Information System. Specific details of data collection were provided elsewhere [5]. Through the Wuhan Statistics Bureau, the permanent resident population of Wuhan in 2018 was 11.081 million. After eliminating the duplicate data, a total of 1833 deaths were included in the study. We removed duplicate data based on the name, ID number and gender. If the name, ID number and gender of the case were the same, then the duplicate data were removed. A total of 59 duplicate entries were excluded before proceeding towards analyses.

### Variables

Demographic data consisted of information on age, sex, occupation, residential area, area of reporting units. Other available data included clinical outcomes, disease severity, report date, date of onset, date of diagnosis, date of death, the interval time from onset to diagnosis, the interval time from diagnosis to death and the interval time from onset to death. The underlying disease variable was determined using patient self-reported history. The severity of symptoms were categorized as mild type, common type, severe type or critical type, the detailed classification criteria were shown in Supplementary Table S1. The crude death rate was estimated as the number of deaths divided by the number of permanent resident population of Wuhan. The date of onset was defined as the day when the symptom was observed.

### Statistical analysis

We present continuous variables as medians (interquartile ranges, IQR) and compared using Mann-Whitney U test. If they are normally distributed, we present continuous variables as mean (Standard Deviation, SD). Categorical variables were described as counts and percentages in each category. The probability distributions of age and interval time were fitted and the distribution with the lowest AIC value was selected as optimal distribution. The map of epidemic situation in Wuhan was drawn based on the number of daily deaths. We used ArcGIS to plot the numbers of daily deaths on maps. Statistical analyses were performed using SPSS and @Risk software.

### Ethics approval

Data collection, which determined by the National Health Commission of the People’s Republic of China, was exempt from institutional review board approval because it was part of outbreak investigation. Study design and data analysis have been reviewed and approved by the Medical Ethical Committees of Wuhan University (WHU2020-2020YF0031).

## Results

### Patients

As of February 24, 2020, a total of 1833 unique deaths with COVID-19 were included in the analysis. Baseline characteristics of deaths are presented in Table 1. According to our database, the first death occurred on January 9, 2020. A majority were aged 50–89 years (89.9%), male (66.1%), retirees (46.7%). Eight health workers died with COVID-19. Among the composition of disease severity, mild type accounted for the largest proportion (37.2%), followed by severe type(30.1%), then 13.6% for critical type and 5.5% for common type.

**Table 1:** The epidemiological characteristics of deceased patients of COVID-19 infection with different severities in Wuhan during the early stage

Baseline characteristics	Total	Classification of severity (%)^**b**^				
		Mild	Common	Severe	Critical	Missing
**Total**	1833	682(37.2)	101(5.5)	551(30.1)	249(13.6)	250(13.6)
**Age**						
*M (IQR)*^a^	70.0 (63.0–79.0)	70.0 (62.0–78.0)	72.0 (64.0–78.5)	71.0 (64.0–80.0)	69.5 (62.0–77.0)	71.0 (62.0–79.0)
**Sex**						
Male	1211(66.1)	438(64.2)	71(70.3)	371(67.3)	155(62.2)	176(70.4)
Female	622(33.9)	244(35.8)	30(29.7)	180(32.7)	94(37.8)	74(29.6)
**Occupation**						
Child and student	2(0.1)	1(0.2)	0(0.0)	1(0.2)	0(0.0)	0(0.0)
Cadre	35(1.9)	14(2.1)	1(1.0)	15(2.7)	4(1.6)	1(0.4)
Freelancer	7(0.4)	2(0.3)	1(1.0)	1(0.2)	3(1.2)	0(0.0)
Physical labor	23(1.3)	15(2.2)	1(1.0)	4(0.7)	3(1.2)	0(0.0)
Public service staff	25(1.4)	14(2.1)	0(0.0)	8(1.5)	2(0.8)	1(0.4)
Housework	324(17.7)	138(20.2)	23(22.8)	105(19.1)	51(20.5)	7(2.8)
Retirees	854(46.7)	343(50.3)	56(55.5)	296(53.7)	129(51.8)	30(12.0)
Farmer or worker	66(3.6)	29(4.3)	4(4.0)	22(4.0)	10(4.0)	1(0.4)
Health worker	8(0.4)	3(0.4)	1(1.0)	3(0.5)	1(0.4)	0(0.0)
Missing	489(26.7)	123(18.0)	14(13.9)	96(17.4)	46(18.5)	210(84.0)
**Underlying diseases**						
Yes	1204(65.7)	430(63.1)	59(58.4)	367(66.6)	172(69.1)	176(70.4)
No	583(31.8)	234(34.3)	38(37.6)	173(31.4)	68(27.3)	70(28.0)
Missing	46(2.5)	18(2.6)	4(4.0)	11(2.0)	9(3.6)	4(1.6)
**Specific Underlying diseases**						
Hypertension						
Yes	742(40.5)	264(38.7)	37(36.6)	231(41.9)	112(45.0)	98(39.2)
No	1045(57.0)	400(58.7)	60(59.4)	309(56.1)	128(51.4)	148(59.2)
Diabetes						
Yes	357(19.5)	133(19.5)	18(17.8)	110(20.0)	50(20.1)	46(18.4)
No	1430(78.0)	531(77.9)	79(78.2)	430(78.0)	190(76.3)	200(80.0)
Cardiovascular disease						
Yes	329(17.9)	119(17.4)	16(15.8)	109(19.8)	36(14.5)	50(20.0)
No	1458(79.5)	545(79.9)	81(80.2)	431(78.2)	204(81.9)	196(78.4)
Respiratory disease						
Yes	152(8.3)	47(6.9)	9(8.9)	57(10.3)	18(7.2)	21(8.4)
No	1635(89.2)	617(90.5)	88(87.1)	483(87.7)	222(89.2)	225(90.0)
Cancer (any)						
Yes	82(4.5)	29(4.3)	5(5.0)	29(5.3)	9(3.6)	10(4.0)
No	1705(93.0)	635(93.1)	92(91.1)	511(92.7)	231(92.8)	236(94.4)
**Date of onset**						
2019.12–2020.1.9	147(8.0)	27(4.0)	5(5.0)	37(6.7)	32(12.9)	46(18.4)
2020.1.10–1.21	525(28.6)	154(22.6)	11(10.9)	149(27.0)	79(31.7)	132(52.8)
2020.1.22–2.1	869(47.4)	391(57.3)	42(41.6)	288(52.3)	107(43.0)	41(16.4)
2020.2.2–2.24	288(15.7)	110(16.1)	43(42.6)	77(14.0)	31(12.4)	27(10.8)
Missing	4(0.2)	0(0.0)	0(0.0)	0(0.0)	0(0.0)	4(1.6)
**District of residence**						
Central urban area	1384(75.5)	574(84.2)	87(86.1)	428(77.7)	199(79.9)	96(38.4)
Surrounding urban area	286(15.6)	102(15.0)	13(12.9)	108(19.6)	41(16.5)	22(8.8)
Out of city	28(1.5)	6(0.9)	1(1.0)	12(2.2)	5(2.0)	4(1.6)
Missing	135(7.4)	0(0.0)	0(0.0)	3(0.5)	4(1.6)	128(51.2)
**Days from onset to death,** ***M (IQR)***	17.0(12.0–22.0)	17.0(12.0–22.0)	16.0(10.0–22.0)	17.0(13.0–23.0)	17.0(12.0–23.0)	16.0(10.0–23.0)
**Days from onset to diagnosis,** ***M (IQR)***	10.0(6.0–14.0)	10.0(6.0–14.0)	9.0(4.0–15.0)	11.0(7.0–15.0)	11.0(7.0–15.0)	10.0(5.0–14.0)
**Days from diagnosis to deaths,** ***M (IQR)***	6.0(2.0–11.0)	6.0(3.0–10.0)	5.0(2.0–10.0)	6.0(3.0–10.0)	6.0(2.0–10.0)	5.0(2.0–12.0)

^a^*M* Medians, *IQR* Interquartile ranges;

^b^ The classification of severity were according to the diagnostic criteria of the new coronavirus infection pneumonia diagnosis and treatment plan (trial fifth version)

### Age distribution and sex ratio

The age of deaths obeyed the Weibull distribution (8.2152, 89.42, − 14.41) illustrated in Table 1. The minimum, maximum and median ages of the deaths were 14.0, 100.0 and 70.0 (IQR: 63.0–79.0), respectively. The number of deaths due to COVID-19 between the ages of 50–89 years was 1647, accounting for 89.9% of the total deaths, and the age distribution of different disease severity was similar.

**Fig. 1:**
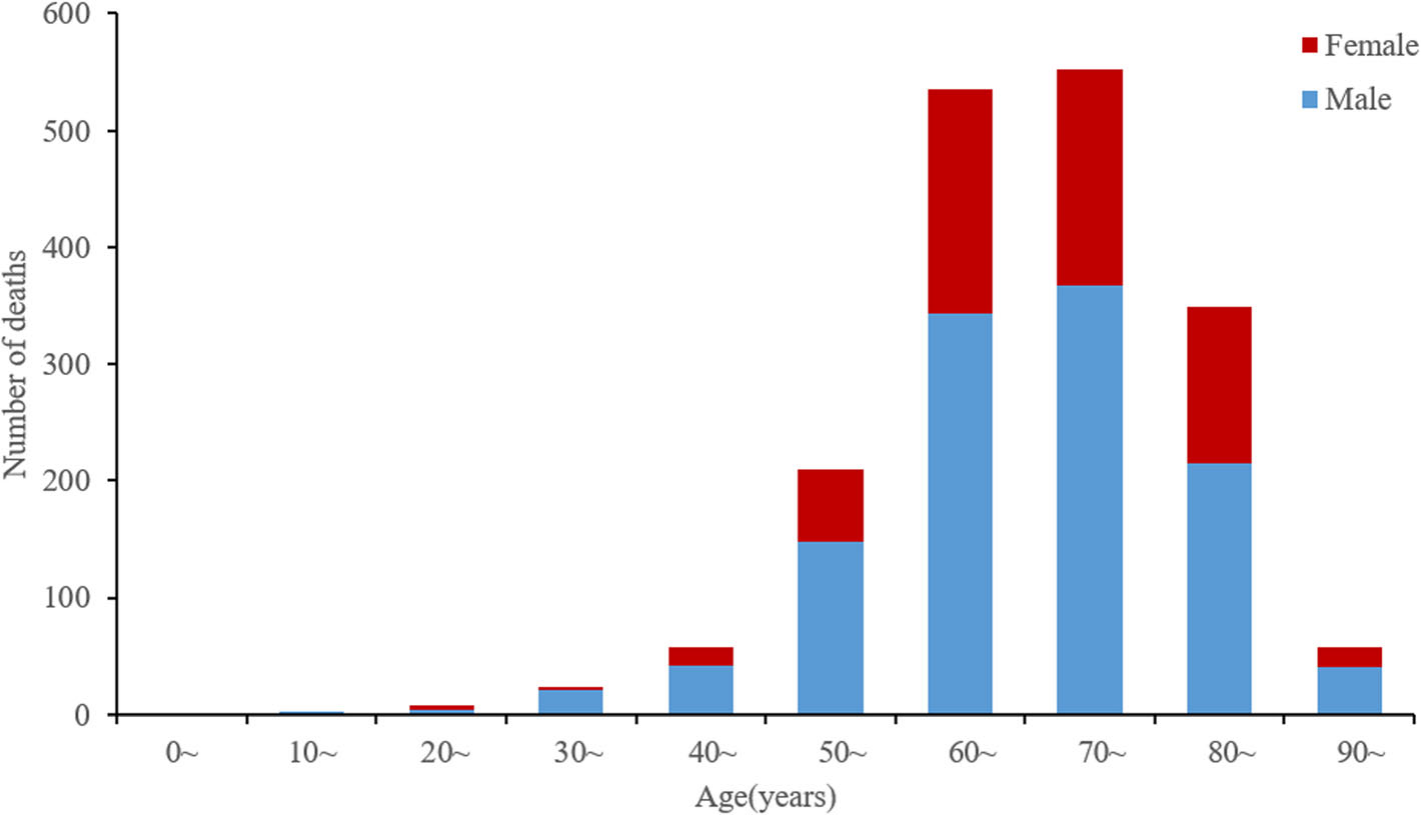
The age distribution of deaths in Wuhan

Among the 1833 deaths, male deaths accounted for 66.1%, and the male to female ratio was 1.95:1 in Wuhan. As shown in Fig. 1, in all age groups, male deaths were significantly more than female deaths. The male to female ratio of different disease severity was similar.

### Underlying diseases

There were 1204 COVID-19 deaths with underlying diseases (65.7%) and 676 deaths with more than one underlying disease (56.2%). Among these underlying diseases, hypertension accounts for the largest proportion—61.6%, then 29.7% for diabetes, 27.4% for cardiovascular diseases, 12.6% for respiratory diseases, and 6.8% for cancer.

The median age of deaths with underlying diseases (M: 72.0 IQR: 64.0–79.0) was higher than that of those who died without underlying diseases (M: 68.0 IQR: 60.0–76.0) (*P* < 0.001). In addition, there were 820 male deaths and 384 female deaths with underlying diseases, the male to female ratio for deaths with underlying diseases was 2.14:1.

### Temporal distribution

**Fig. 2:**
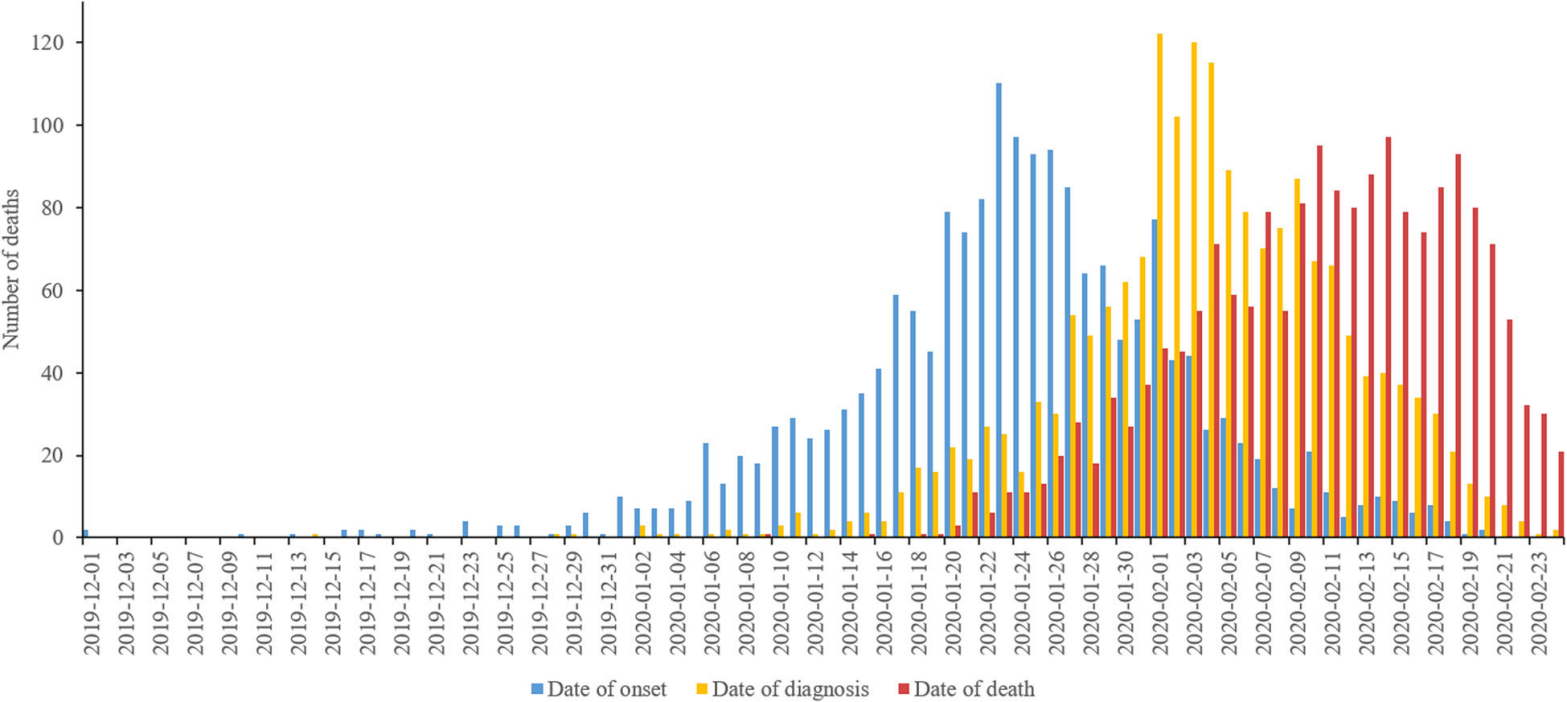
The epidemiological curves by date of symptom onset, date of diagnosis and date of death in Wuhan

Figure 2 shows the COVID-19 epidemic curve with number of deaths plotted by date of patient’s onset of symptoms, diagnosis and death. The peak onset of symptoms for COVID-19 deaths occurred on January 23, 2020. Since then, onset of illness has declined. The number of diagnosis reached the peak of 122 on February 1, 2020, and the daily deaths reached the peak of 97 on February 14.

**Fig. 3:**
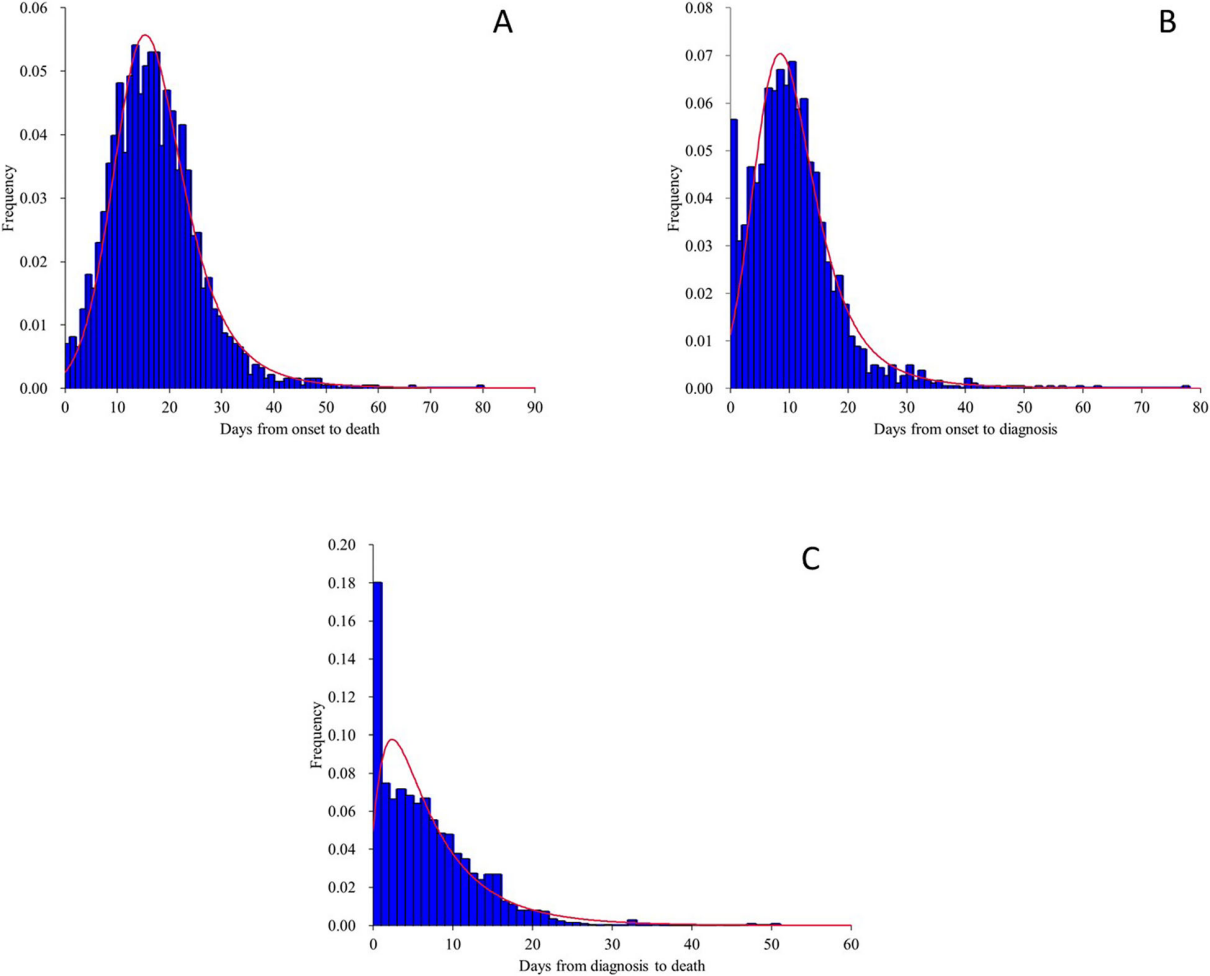
Time distribution of interval from onset to death (**a**), interval from onset to diagnosis (**b**) and interval from diagnosis to death (**c**)

As shown in Fig. 3. The median interval from symptom onset to diagnosis was 10.0 (IQR: 6.0–14.0) days and obeyed the Log Logistic distribution. Moreover, for those whose onset date was before January 15, between January 15–31, and after January 31, the median intervals from onset to diagnosis were 15.0, 11.0, and 5.0 days, respectively. The interval from onset to diagnosis gradually shortened, the efficiency of diagnosis is improving. In addition, The median of interval from onset to diagnosis for 746 cases before January 23 was 13.0 days (IQR: 10.0–18.0), which was significantly longer than the 1058 cases after January 23 with a median of 8.0 days (IQR: 5.0–12.0) (*p* < 0.001). The median interval from diagnosis to death was 6.0 (IQR: 2.0–11.0) days and obeyed the InvGauss distribution. The median interval from symptom onset to deaths was 17.0 (IQR: 12.0–22.0) days and obeyed the Log Logistic distribution. The distribution fit is shown in Table 2.

**Table 2:** Distributional fits to key COVID-19 distributions

Variable	Distribution	Parameter1	Parameter2	Parameter3	Median	25%	75%
**Age**	Weibull	8.2152	89.42	−14.41	71.1	62.4	78.6
**Days from onset to death**	Log Logistic	−14.513	31.184	6.8020	16.7	12.0	22.1
**Days from onset to diagnosis**	Log Logistic	−7.7179	17.669	4.7575	10.0	6.3	14.5
**Days from diagnosis to deaths**	InvGauss	9.8986	19.1904	−2.4969	5.4	2.6	10.0

### Geographical distribution

A total of 13 administrative regions constitute Wuhan, of which Jiang’an District, Jianghan District, Qiaokou District, Hanyang District, Wuchang District, Qingshan District and Hongshan District are the central urban area, Dongxihu District, Hannan District, Caidian District, Jiangxia District, Huangpi District and Xinzhou District are the surrounding urban area.

**Fig. 4:**
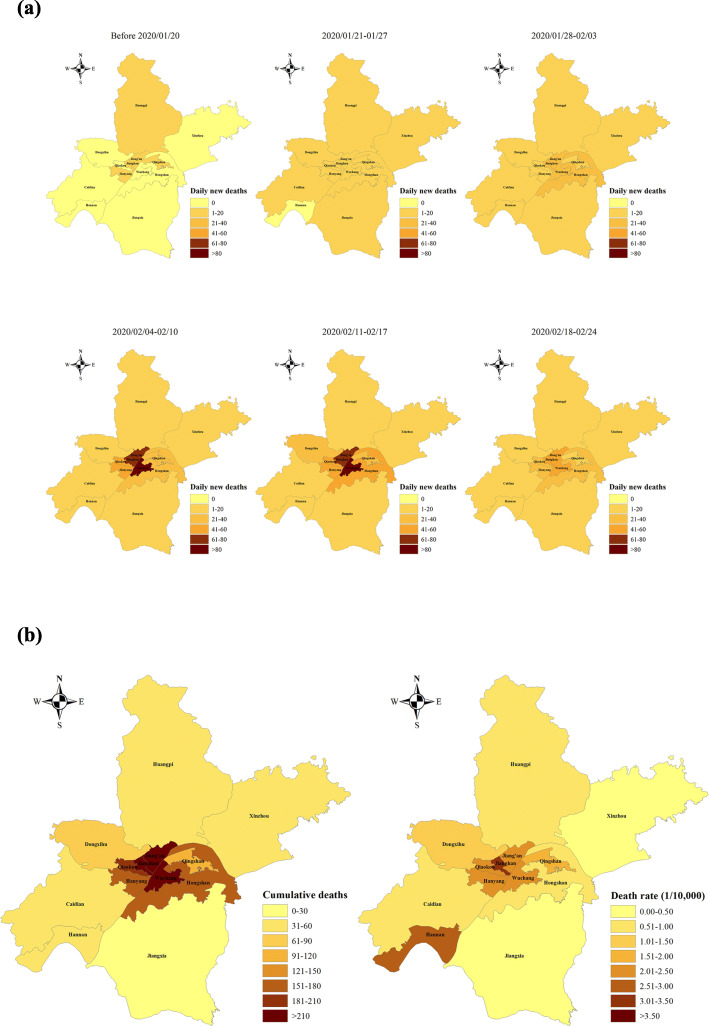
**a** The geographic distribution of daily new COVID-19 death in administrative districts of Wuhan (2020/01 / 09–2020 / 02/24). **b** The geographic distribution of cumulative deaths and death rate in Wuhan

Figure 4 showed the geographical distribution of daily new COVID-19 deaths in Wuhan. The deaths were few in early January 2020, and gradually increased in late January. The epidemic situation in Wuhan was serious from the end of January to mid February, and then the number of daily deaths in each district gradually decreased in the late February.

Meanwhile, the geographical map showed that the COVID-19 epidemic situation in Wuhan had obvious regional differences. The epidemic situation in the central urban areas represented by Jianghan District were relatively serious, while the surrounding urban areas were relatively mild. As shown in Table 3, as of February 24, sort by residence, the top 3 regions in terms of death rate were 3.26‱ in Jianghan District, 2.65‱ in Hannan District and 2.47‱ in Jiang’an District. According to the medical facility where the death was, the top 3 regions ranked by death rate were 6.48‱ in Hannan District, 4.26‱ in Dongxihu District and 2.46‱ in Qingshan District.

**Table 3:** Death rate of COVID-19 cases in administrative districts of Wuhan

District	Permanent population (10,000)	Residence		Reporting Unit area	
		Deaths, N	Death rate per 10,000 residents	Deaths, N	Death rate per 10,000 residents
Caidian	76.16	43	0.56	153	2.01
Dongxihu	58.48	79	1.35	249	4.26
Hannan	13.58	36	2.65	88	6.48
Hanyang	66.42	161	2.42	112	1.69
Hongshan	167.73	159	0.95	164	0.98
Huangpi	101.19	58	0.57	54	0.53
Jiang’an	96.27	238	2.47	186	1.93
Jianghan	72.97	238	3.26	170	2.33
Jiangxia	96.20	29	0.30	32	0.33
Qiaokou	86.87	181	2.08	167	1.92
Qingshan	52.89	103	1.95	130	2.46
Wuchang	128.28	299	2.33	303	2.36
Xinzhou	91.06	41	0.45	25	0.27

As of February 24, the cumulative deaths in the central urban area accounted for 82.8% of the total deaths. The death rate of the central urban areas (2.05‱) was heavier than that of the surrounding areas(0.65‱). Due to the small population in Hannan District, the death rate was relatively high.

## Discussion

Wuhan is the capital of Hubei Province and a megacity in central China. After the COVID-19 epidemic occurred in December 2019, strong prevention and control measures were taken to prevent the epidemic from spreading. Based on the stochastic transmission model, Adam J Kucharski showed that the median daily reproduction number in Wuhan declined from 2.35 (95%CI: 1.15–4.77) per week before travel restrictions were introduced on Jan 23, 2020, to 1.05 (95%CI: 0.41–2.39) per week after [6]. Research by Wang Xuyan showed that the COVID-19 epidemic in Hubei Province has gradually eased from mid to late February, and the prevention and control measures were very effective [7]. As of February 24, 2020, the number of daily deaths in Wuhan has shown a clear downward trend.

In the composition of deaths, mild type accounted for the largest proportion, 37.2%, followed by severe type deaths, accounting for 30.1%, and the initial diagnosis of the dead with underlying diseases is mostly mild type, so we should be alert to the deterioration of mild type.

Judging from the age distribution of diagnosed cases across the country, people of all ages are not resistant to the COVID-19 [5]. The analysis of the expert group also supported this view [8]. Older people and those with underlying diseases such as asthma, diabetes and heart disease may be at increased risk of infection [9]. In our study, 65.7% of the deaths in Wuhan combined with underlying diseases, the main combined underlying diseases were hypertension, diabetes and cardiovascular diseases. Studies have shown that combination with underlying diseases such as hypertension, diabetes and cardiovascular disease may increase the mortality rate of COVID-19 patients [10–12]. It may be because the metabolic syndrome can downregulate the key mediator of the host’s innate immune response to pathogenesis, affecting the function of the innate and humoral immune systems [13]. In addition, the pathogen of COVID-19, SARS-COV-2, is mainly bound to target cells by angiotensin-converting enzyme 2(ACE2) and ACE1 drugs are often used in cardiovascular disease patients, and long-term use of ACE1 can up-regulate the expression of ACE2 receptors in the body [14]. Thiazolidinedione in hypoglycemic drugs can also cause upregulation of ACE2 expression [15], which may aggravate the patient’s symptoms.

The deaths in Wuhan were mainly middle-aged and elderly people, mainly concentrated in the age group of 50–89 years. It may be due to the weaker physical resistance of the middle-aged and elderly people and the higher probability of combining the underlying diseases. The minimum age of deaths is 14 years old, and no 0–9 years old deaths occurred. SARS also has a low impact on children, considering the commonness of coronaviruses, children may be relatively unsusceptible to COVID-19 based on cellular structure or immunity [16]. There are no reports of COVID-19 causing maternal and infant deaths. In addition, the possibility of vertical transmission of coronavirus is very low. There are no recorded cases of vertical transmission of SARS or MERS [17, 18], and the COVID-19 has not been confirmed vertical transmission [19–21].

The male to female ratio of confirmed cases issued by the China CDC was 0.99: 1 in Wuhan and 1.06: 1 in China overall [5], which indicated that men and women are equally susceptible to COVID-19. However, the fact that the sex ratio of deaths in Wuhan is 1.95: 1 showed male patients have a higher risk of death. As coronaviruses, SARS-CoV and COVID-19 have similar sex differences, probably because the X chromosome and estrogen can protect women from fatal infections [22, 23]. Our findings content with many reports that indicated being male is highly associated with death due to COVID-19. Men were at a higher risk of death due to COVID-19 in India [24], the USA [25] and Brazil [26]. But in Nepal, female had a higher risk of death, this may because women in Nepal have higher smoking prevalence and higher risk of suffering from non-communicable diseases [27].

Among all occupational categories, the number of retirees’ deaths was the largest, accounting for 46.7%. And health workers often have close contact with patients during treatment, nursing, accompanying and visiting patients, the risk of nosocomial infection is high. Among the deaths, 8 health workers died with COVID-19, accounting for 0.4% of the total deaths. Children and students accounted for the least percentage of deaths, at 0.1%. Studies have shown that the SARS-CoV-2 can use multiple homologous genes of angiotensin converting enzyme II (ACE2) to effectively replicate in human respiratory tract cells [28]. The relatively low incidence of child deaths may be due to the relatively weak function of ACE2 receptors in children, or low expression, which limits the path of viral invasion and avoids large-scale outbreaks in children [16].

Analysis of 1833 deaths in Wuhan found that the mean interval from onset to diagnosis was 11.2 days; the median time was 10.0 days. Moreover, the interval from onset to diagnosis gradually shortened. For deaths with onset before January 15, between January 15–31 and after January 31, the median interval from onset to diagnosis is 15.0 days, 11.0 days and 5.0 days, respectively, indicating that the ability to discover and diagnose COVID-19 cases has gradually improved. Since January 23, Wuhan has adopted many epidemic prevention and control measures. After January 23, the median of interval from onset to diagnosis were reduced from 13.0 days to 8.0 days, which was significantly shorter than that before January 23. This indicates that the early identification, isolation and confirmation of cases with COVID-19 have been accelerated in Wuhan. Shortening the duration of onset to diagnosis facilitates quarantine and reduces the risk of transmission, and the effective communicable period.

In addition, according to reports from India and Nepal, 1042 fatal cases (18.2% of 5733 observed) were identified ≤24 h before death or posthumously in Tamil Nadu and Andhra Pradesh [24] and more than half of the individuals who died were diagnosed as SARS-CoV-2-positive after death in Nepal [27]. Our database didn’t support this analysis. However, according to the revised fifth version of the guideline over the diagnosis and treatment of COVID-19 [29], Clinical diagnosis was being used in Hubei Province only (China) form February 8 to February 19. Clinically diagnosed cases were diagnosed by symptoms, exposures and CT scan only. Clinical diagnosis was effective, which is helpful to quarantine or treat infected cases as soon as possible, and prevent the epidemic from worsening.

According to the death data as of February 24, the first case of COVID-19 death in Wuhan was located in Huangpi District. The geographical map showed the distribution of COVID-19 epidemic in Wuhan had obvious regional differences. The central urban area was more serious than the surrounding urban areas. The cumulative deaths reported in the central urban area accounted for 82.8%. The death rate of COVID-19 in Wuhan was also the highest in the central urban area. This regional difference may be due to the fact that the Huanan Seafood Wholesale Market, the outbreak point, was located in Jianghan District, and the traffic in the central urban area was convenient, and the designated COVID-19 hospitals in the early stage of the epidemic were also located in the central urban area.

This study also has some limitations. First, some variables in this study have missing records, which may slightly affect the results. Second, the data on pre-existing underlying diseases were based on self-report and we could not verify their validity. Third, the onset date of this study was obtained from the patient’s self-report, and there may be a recall bias.

In summary, the COVID-19 posed a great threat to the elderly, especially elderly men with underlying diseases living in non-central areas. The geographical distribution showed that the epidemic in the central area of Wuhan was more serious than that in the surrounding areas. The reduction in the interval from onset to diagnosis indicated a gradual improvement of the detection and diagnosis ability. The number of daily deaths in Wuhan had continued to decline after February 14, indicating that the COVID-19 epidemic in Wuhan had achieved a tremendous improvement, and the strong epidemic control measures taken by Wuhan Government were very effective. Based on the above discussion, to further control the epidemic, it is necessary to carry out key monitoring, prevention and control of the elderly men, and strengthen early warning and intervention of severe and critical cases. Provide assistance to areas where health resources were relatively or limited. Prevent hospital and health workers infections along with proper disposal of hospital wastes. Improving the detection and treatment capacity of hospitals and isolating the source of infection in time would be better options to control the disease spread.

## Supplementary Information

**Additional file 1:** **Table S1.** The classification criteria of severity of COVID-19.

## Data Availability

The data that support the findings of this study are available from China CDC but restrictions apply to the availability of these data, which were used under license for the current study, and so are not publicly available. Data are however available from the authors upon reasonable request and with permission of China CDC.
