# Correction to: The epidemiological characteristics of deaths with COVID-19 in the early stage of epidemic in Wuhan, China

**DOI:** 10.1186/s41256-020-00184-x

**Published:** 2020-12-31

**Authors:** Jianjun Bai, Fang Shi, Jinhong Cao, Haoyu Wen, Fang Wang, Sumaira Mubarik, Xiaoxue Liu, Yong Yu, Jianbo Ding, Chuanhua Yu

**Affiliations:** 1 Department of Epidemiology and Biostatistics, School of Health Sciences, Wuhan University, 115#Donghu Road, Wuhan, 430071 China; 2 School of Public Health and Management, Hubei University of Medicine, 30# South Renmin Road, Shiyan, 442000 China; 3 YEBIO Bioengineering Co., Ltd. of Qingdao, 21# Aodongnan Road, Qingdao, 266114 China; 4 Global Health Institute, Wuhan University, 185# Donghu Road, Wuhan, 430072 China

**Correction to: Glob Health Res Policy (2020) 5:54**

**https://doi.org/10.1186/s41256-020-00183-y**

After publication of this article [1], it is noticed the format of author names in reference 7 is incorrect.

The correct reference 7 is below:

Wang X, Yu Y, Hu Y, Yu C. COVID-19 analysis and forecast based on exponential smoothing model in Hubei Province. J Public Health Prev Med. 2020;31(1):1–4. (in Chinese)

The original article has been updated.

## References

[1] Bai J, Shi F, Cao J (2020). The epidemiological characteristics of deaths with COVID-19 in the early stage of epidemic in Wuhan, China. Glob Health Res Policy.

