# Supplementary material for: The epidemiological characteristics of deaths with COVID-19 in the early stage of epidemic in Wuhan, China
Source: Glob Health Res Policy. 2020 Dec 21;5:54. doi: 10.1186/s41256-020-00183-y (PMC7750392; doi:10.1186/s41256-020-00183-y)
Supplement: Supplementary file 1 — Additional file 1: Table S1. The classification criteria of severity of COVID-19. [file 41256_2020_183_MOESM1_ESM.docx]

**Supplementary Table S1**. The classification criteria of severity of COVID-19

| Classification | Criteria |
| --- | --- |
| Mild type | The clinical symptoms were mild, and no pneumonia was found on the chest computed tomography(CT) |
| Common type | Fever, respiratory symptoms, and patients found to have imaging manifestations of pneumonia |
| Severe type | Fulfilling one of the following three conditions: Respiratory distress, respiratory rate ≥ 30times/min (in resting state, refers to oxygen saturation ≤ 93%), partial arterial oxygen pressure (PaO2)/oxygen absorption concentration (FiO2) ≤ 300 mmHg (1 mmHg = 0.133 kPa) |
| Critical type | Fulfilling one of the following three conditions: Respiratory failure and the need for mechanical ventilation, shock, or the associated failure of other organs requiring the intensive care unit |
